# Decline and Local Extinction of Caribbean Eusocial Shrimp

**DOI:** 10.1371/journal.pone.0054637

**Published:** 2013-02-13

**Authors:** J. Emmett Duffy, Kenneth S. Macdonald III, Kristin M. Hultgren, Tin Chi Solomon Chak, Dustin R. Rubenstein

**Affiliations:** 1 Virginia Institute of Marine Science, The College of William and Mary, Gloucester Point, Virginia, United States of America; 2 Department of Fish, Wildlife and Conservation Ecology, New Mexico State University, Las Cruces, New Mexico, United States of America; 3 Department of Biology, Seattle University, Seattle, Washington, United States of America; 4 Department of Ecology, Evolution and Environmental Biology, Columbia University, New York, New York, United States of America; The Australian National University, Australia

## Abstract

The tropical shrimp genus *Synalpheus* includes the only eusocial marine animals. In much of the Caribbean, eusocial species have dominated the diverse fauna of sponge-dwelling shrimp in coral rubble for at least the past two decades. Here we document a recent, dramatic decline and apparent local extinction of eusocial shrimp species on the Belize Barrier Reef. Our collections from shallow reefs in central Belize in 2012 failed to locate three of the four eusocial species formerly abundant in the area, and showed steep declines in colony size and increases in frequency of queenless colonies prior to their disappearance. Concordant with these declines, several nonsocial, pair-forming *Synalpheus* species increased in frequency. The decline in eusocial shrimp is explained in part by disappearance of two sponge species on which they specialize. Eusocial shrimp collections from Jamaica in 2012 showed similar patterns of decline in colony size and increased queenlessness compared with prior Jamaican collections. The decline and local extinction of eusocial shrimp happened against a backdrop of changes in coral assemblages during recent decades, and may reflect changes in abundance and quality of dead coral substratum and succession of the diverse cryptic organisms living within it. These changes document potentially worrisome declines in a unique taxon of eusocial marine animals.

## Introduction

Tropical reefs are widely recognized as centers of marine biodiversity [1]. The corals that build them have therefore been well studied, as have the charismatic and ecologically important fishes they support. But within the porous reef structure of dead corals, algae, mollusks, and other organisms lives a rich variety of largely invisible organisms of every phylum. Although most of these species are poorly known they comprise the bulk of marine biodiversity.

A prominent group within this cryptic reef community is the alpheid or snapping shrimps and particularly the genus *Synalpheus*, which contains >150 described species worldwide and many more that are undescribed [2]. In the West Atlantic, most species of *Synalpheus* belong to the informal “gambarelloides” group, which are obligate sponge inhabitants that live within their hosts’ internal circulatory canals and feed on sponge tissue [3], [4]. Most of these sponge-dwelling *Synalpheus* are highly host-specific, living in one or a few species of sponges [5]. Some sponge species house only a single species of *Synalpheus*, whereas other sponges can support multiple shrimp species, sometimes even simultaneously [6]. *Synalpheus* shrimp are of particular interest because some of the species (about seven of the ∼40 West Atlantic sponge-dwellers) are eusocial, indeed the only known eusocial marine animals. That is, while most *Synalpheus* species form heterosexual pairs or live in aggregations of pairs, eusocial shrimp live in colonies of tens to hundreds of individuals, generally with a single reproductive female, or “queen” [7], [8]. On many Caribbean reefs, the eusocial species dominate the *Synalpheus* shrimp fauna, and indeed are among the most abundant macroscopic animals. Comparative studies in Belize showed that eusocial *Synalpheus* species were more abundant, used a wider range of host species, and were less likely to share the host with congeners, than were their less social relatives [6]. This dominance by eusocial species may be facilitated by the benefits of cooperative nest defense [9]. Whatever the reason, eusocial species have dominated the diverse faunas of cryptic sponge assemblages in several Caribbean areas since our collections began nearly a quarter century ago [5], [10], [11].

In 2012 we revisited Carrie Bow Cay, Belize as part of a long-term research program [7], [12], [13]. To our surprise the eusocial shrimp species that have dominated reefs at this site for >20 years had apparently disappeared. Here we document this temporal change by assembling data on the changing species composition and abundance of cryptic sponges and their associated shrimps. We also document changes in the structure of eusocial shrimp colonies in Belize over the past 16 years, and present preliminary evidence for similar patterns in Jamaica.

## Methods

### Sampling

Our analysis is based on specimens of shrimp (*Synalpheus* spp.) and sponges collected in Belize and Jamaica. In Belize, we sampled cryptic reef sponges and associated shrimp from dead coral rubble in 1–3 m depth in the Sand Bores region, a collection of small reefs and banktops that rise from the sand-bottomed lagoon behind the Barrier Reef in the vicinity of the Smithsonian Institution’s field station at Carrie Bow Cay (16°48.155′N, 088°04.896′W). Between 1996 and 2009, samples of reef rubble were collected on 21 separate days; in 2012 we sampled the Sand Bores region systematically and intensively to compare the communities with those of prior years, collecting ten bags of coral rubble (1.2–7.1 liters each, median 3.6 L) from each of six reefs spanning the geographic extent of the Sand Bores complex (see [5] for more detailed methods).

Most sponges in the Sand Bores were cryptic, filling spaces among eroded branches of dead coral. In the field, pieces of dead coral rubble were collected, placed in mesh bags, transported back to the station at Carrie Bow Cay, and retained in flowing seawater until they could be processed. Rubble was broken apart and all sponges were provisionally identified. Sponges were then dissected and macrofauna was removed from the internal canals of the sponge. *Synalpheus* shrimps were sorted by species, counted, then preserved in Davidson’s fixative, 10% formalin in seawater, 70% EtOH, or 95% EtOH. Representative samples of each sponge species were preserved in 70% EtOH for later identification.

In Jamaica we made two collections, one in January 2008 and one in January 2012. In 2008 our collections were exploratory and intended to capture as much of the *Synalpheus* diversity as possible [11]. In 2012 our primary goal was to locate colonies of eusocial shrimp species. Because we have data from only two collections, and since sampling was not quantitative in either year, we focus here on changes in the structure of Jamaican eusocial shrimp colonies rather than trends in sponge or shrimp community composition.

Analysis of temporal change in sponge-dwelling shrimp communities is constrained by the highly heterogeneous nature of the coral rubble habitat, which makes quantitative sampling challenging, and by the haphazard nature of sampling this material during the early years. However, the Sand Bores collections prior to 2012 were intended to maximize shrimp diversity, and the cryptic sponges were often not visible prior to collection, hence we have no reason to suspect that our collections were strongly biased relative to natural abundances or species composition. Nevertheless, to minimize the influence of haphazard collections or variation in sampling effort, we pooled all specimens collected on a given day for the pre-2012 collections, treating the aggregated specimens from a given day as a single sample. In 2012 we collected larger, more systematic samples consisting of bags of rubble and sponges; each such bag is considered a separate sample in 2012.

### Identification

Vouchers of most sponge species were kindly identified by Dr. Klaus Ruetzler at the US National Museum of Natural History. Provisional identification of *Synalpheus* species in the field was based on observations of the color, host associations, body size, and social structure of living shrimp, and confirmed by careful microscopic examination of morphology according to the latest taxonomy of the group [11], [13]–[17], as well as molecular sequence data in some cases [18].

### Community Analysis

We characterized community structure, separately for *Synalpheus* shrimps and sponges, using Non-metric MultiDimensional Scaling (NMDS), a non-parametric ordination approach, implemented in PRIMER [19]. For NMDS analysis of shrimp communities, we used relative abundances (% of total shrimp in sample) rather than raw abundance as input to control for differences in sampling effort among days; relative abundances were square-root transformed prior to analysis to reduce the influence of dominant species. For analysis of sponge community composition, we used presence/absence data as input because (i) some species of sponges enmeshed in the coral rubble could rarely be collected without breaking them into several pieces, such that abundance could not be accurately estimated, and (ii) sponge sizes were not measured in most collections. For each analysis we constructed a resemblance matrix among samples based on Bray-Curtis similarity measures, and generated the NMDS plots using 100 random starts and the best 2D solution. To estimate the goodness of fit of the NMDS ordination to the data, we calculated stress, i.e., the mismatch between the observed rankings in similarity among samples and the distance rankings estimated from the ordination; stress values <0.20 are generally considered “useful” [19]. We tested differences among groups (*time period*: before 2012 vs. 2012) using the analysis of similarity (ANOSIM) procedure in PRIMER, and the SIMPER procedure to identify the contributions of individual species to differences between groups. Analyses used 999 permutations; significance values are pseudo-*p* values.

## Results

### Changes in Sponge and Shrimp Communities Through Time

**Figure 1:**
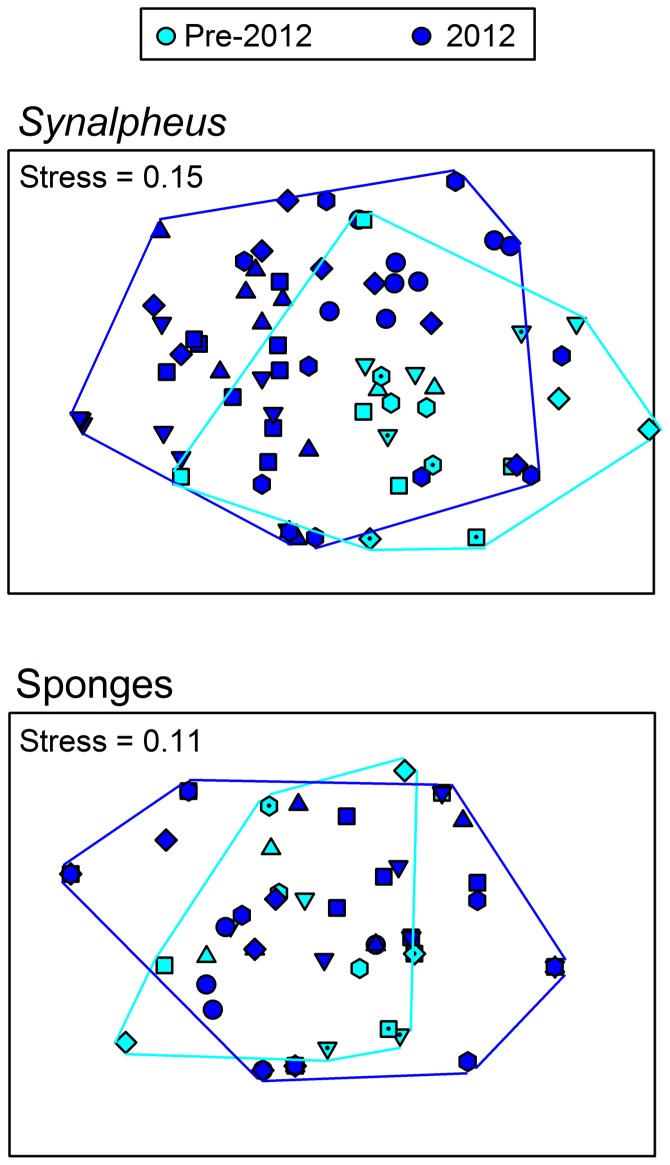
Changes in (A) shrimp and (B) sponge community composition through time. Non-metric Multidimensional Scaling (NMDS) was performed on square-root transformed relative abundances of shrimp, and on presence/absence data for sponges. Light blue symbols and lines represent samples from pre-2012, whereas dark blue symbols and lines are samples from 2012.

**Figure 2:**
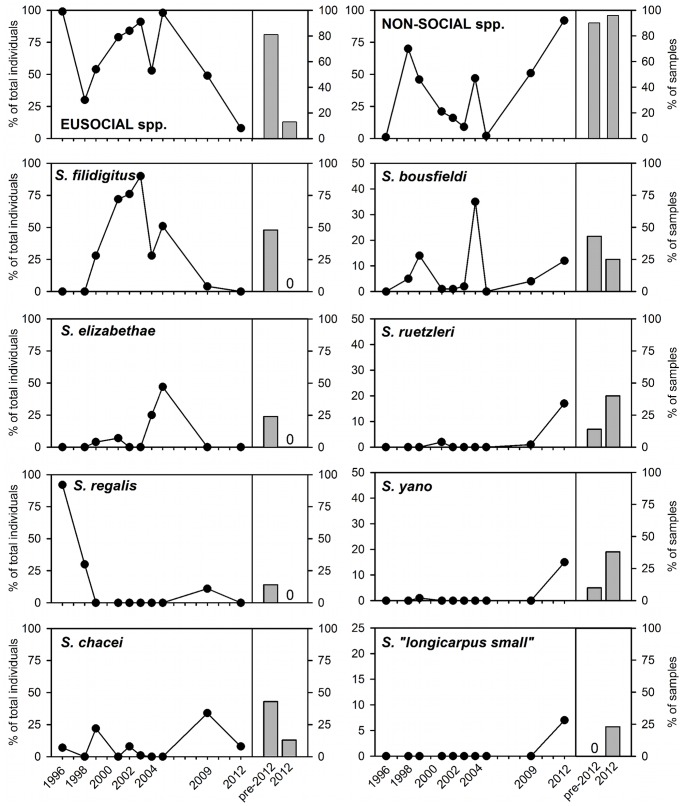
Changes in relative abundance of (left) eusocial and (right) nonsocial, pair-forming shrimp through time in Belize. The left part of each panel shows the percentage of the total sampled shrimp made up by the focal species in collections from that year; gray bars at right show the percentage of samples in which the species was found before 2012 versus in 2012. The top panels shows relative abundance of all eusocial (left) and non-social (right) species aggregated together; remaining panels show individual species. Sample sizes (number of days sampled in collections before 2012, number of sampled bags in 2012) were: 1996 (1), 1998 (1), 1999 (3), 2001 (3), 2002 (2), 2003 (2), 2004 (2), 2005 (2), 2009 (5), 2012 (60).

Composition of the Belize shrimp assemblage in 2012 differed significantly from that in prior years (Fig. 1, P<0.001, ANOSIM), the differences largely reflecting absence in 2012 of eusocial shrimp species. NMDS of the shrimp assemblage, in this case of square root-transformed abundances, produced an acceptable ordination (stress = 0.15), and the pattern of difference among time periods appeared robust since a second NMDS analysis conducted on presence/absence of shrimp species in each sample produced a similar pattern (Fig. S1) with stress = 0.14. The changing nature of *Synalpheus* assemblages resulted from opposite trends through time for eusocial versus nonsocial species. Specifically, SIMPER revealed that the strongest contributors to changing shrimp assemblages through time were the eusocial species *S. filidigitus, S. chacei,* and *S. regalis*, as well as the nonsocial pair-forming species *S. bousfeldi*, which together accounted for 45% of the dissimilarity between collections in 2012 and in prior years. Most notable was the eusocial shrimp *S. filidigitus*, a dominant species on shallow reefs in the Sand Bores, which occurred in roughly half of the samples and comprised 54% of all shrimp collected prior to 2012, but was completely absent in 2012. The decline of *S. filidigitus* illustrates a general trend among eusocial species: across the 21 samples from the Sand Bores between 1996–2009, the four eusocial species *S. filidigitus*, *S. regalis, S. elizabethae*, and *S. chacei* comprised 54%, 16%, 6%, and 6% of total shrimp collected, respectively, together accounting for over 80% of shrimp collected from shallow reefs during that decade (Fig. 2, Table S1). Although the relative abundances of eusocial species varied through time, the pooled abundance of eusocial shrimp remained high for most of the period (Fig. 2). By 2009 the eusocial species *S.filidigitus* and *S. regalis* were rare, represented by two colonies each, and *S. elizabethae* was not found in 2009. In 2012 none of these eusocial species was present in any of the 60 samples, despite the collection spanning a larger area than in any previous year.

Other strong contributors to temporal change in the shrimp assemblage were pair-forming and species and species that live in small, non-eusocial groups, which showed the opposite pattern (Fig. 2): *S. ruetzleri, S. yano*, and *S*. “*longicarpus* small” were relatively rare prior to 2012, but were dominant species in 2012. *S. bousfieldi*, a socially plastic species that usually lives in pairs or small groups, fluctuated strongly among dates with little discernible pattern in abundance or group size. The trend of several pair-forming species increasing in abundance as eusocial species declined partly reflects the fact that percentages are not independent of one another. However, frequencies of occurrence, which are not correlated in this way, show a similar pattern, namely increased commonness of the pair-forming species *S. ruetzleri, S. yano*, and *S. “longicarpus small”* in 2012 as the eusocial species disappeared (Fig. 2 right-hand panels, Table S1).

In contrast to the shift in shrimp assemblages, composition of sponge assemblages showed little change in 2012 compared with prior years (Fig. 1, P = 0.125, ANOSIM). NMDS of the sponge community produced an ordination with relatively low stress (0.11), indicating a good fit. Despite the lack of significant change in overall composition of the sponge assemblage, however, our extensive collections from 2012 located no specimens of the favored hosts of eusocial shrimp *S. regalis* and *S. filidigitus* in the Sand Bores reefs, namely *Neopetrosia* (formerly *Xestospongia*) *proxima*, *N*. cf. *subtriangularis*, or *Oceanapia* sp. These sponges collectively occurred in 11 of 21 samples prior to 2012 but were completely absent in 2012 (Table S2).

### Changes in Eusocial Shrimp Colony Structure Through Time

**Figure 3:**
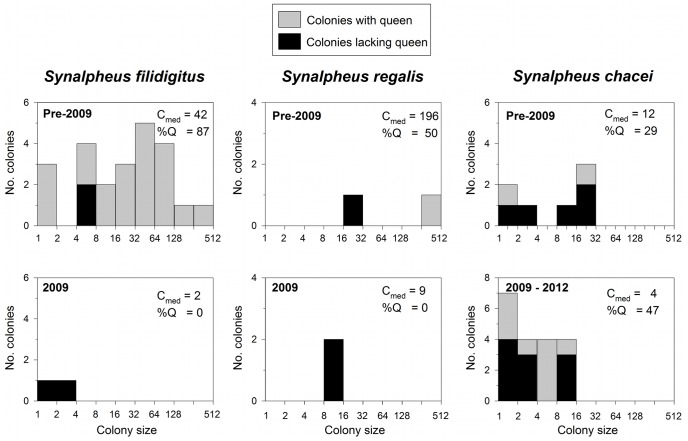
Changes in shrimp colony size and frequency of queenless colonies in Belize through time. Queenless colonies are shown as the black portion of each bar. C_med_ = median colony size (number of individual shrimp per sponge), %Q = the percentage of colonies that contained a breeding queen.

The declining trend in commonness of eusocial shrimp colonies in Belize coincided with parallel strong declines in average colony sizes and increases in the frequency of queenless colonies (Fig. 2). The trend is dramatically illustrated by *S. filidigitus* (Fig. 3): between 1996 and 2005 we collected 23 colonies of *S. filidigitus* with a median size of 42 individuals, all but three of which (87%) included a queen, whereas in 2009 we found only two colonies, each with two individuals and neither with a queen. In 2012, our extensive search of the Sand Bores region yielded no *S. filidigitus*. The highly social species *S. regalis* was uncommon in the shallow Sand Bores, having been historically more common on deeper reefs, but showed a similar pattern: prior to 2009, we collected two *S. regalis* colonies of 19 and 374 individuals from the Sand Bores, the smaller of which was queenless, whereas in 2009 we collected only two colonies, each with nine individuals and no queen. We found no *S. regalis* in the 60 samples collected from the Sand Bores in 2012. The eusocial species *S. elizabethae* was not found in 2009 or 2012 so we could not compare colony sizes through time for this species.

The one partial exception to the trend of declining eusocial shrimp in Belize was *Synalpheus chacei*, which remained present in 2012, albeit less frequent (Fig. 2, Table S1). *S. chacei* is a comparative generalist that uses several species of sponges as hosts in Belize [5] and elsewhere. *S. chacei* showed no clear change in either abundance or colony size between 2012 and prior years (Fig. 2, 3). Although the trend in *S. chacei* was also toward smaller colonies in the later collections, this was not as marked as in the other eusocial species.

### Similar Changes in Jamaican Shrimp Communities

**Figure 4:**
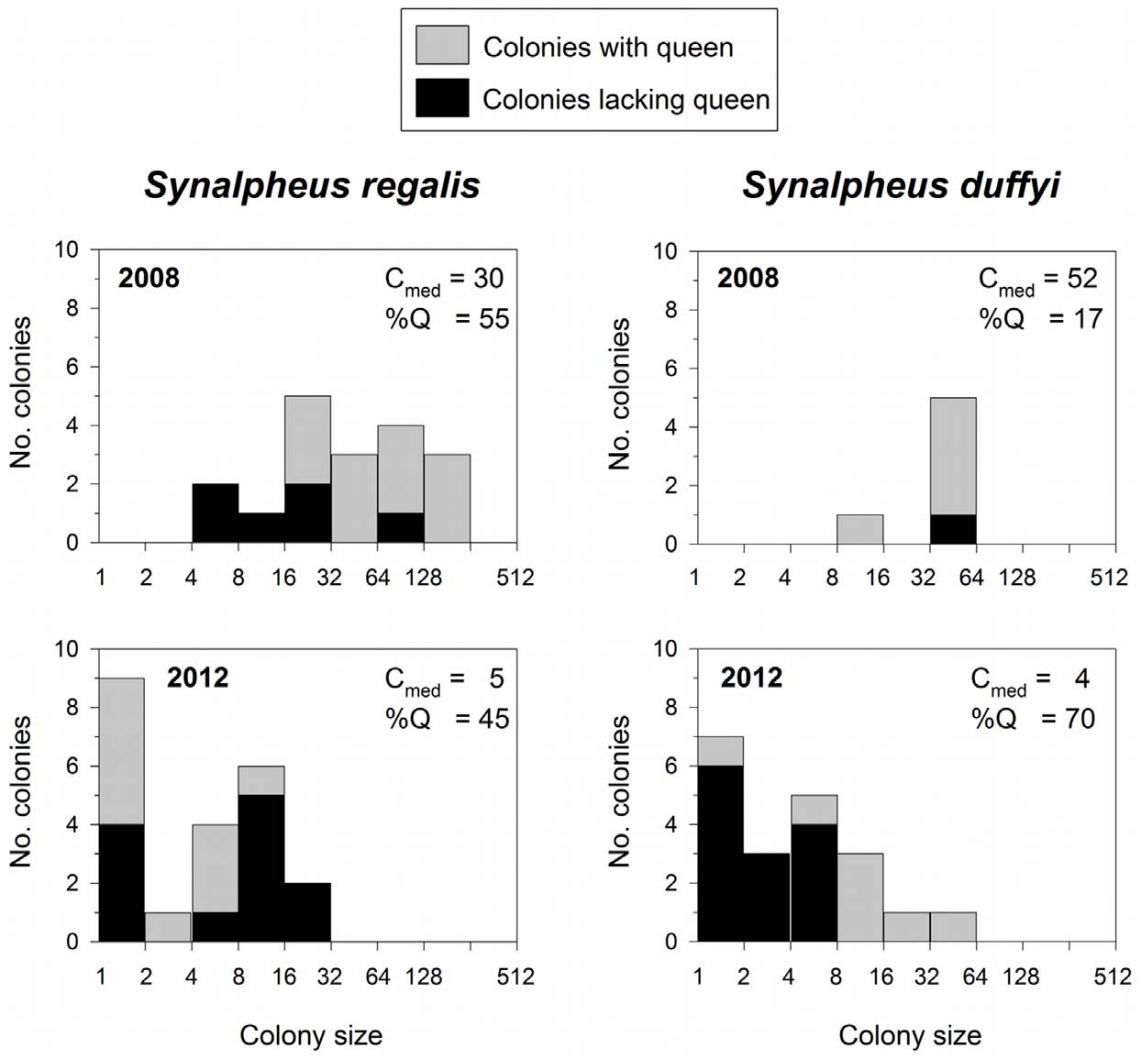
Changes in shrimp colony size and frequency of queenless colonies in Jamaica through time. Queenless colonies are shown as the black portion of each bar. C_med_ = median colony size (number of individual shrimp per sponge), %Q = the percentage of colonies that contained a breeding queen.

In Jamaica, we made collections in only two years but these produced a larger number of colonies than for several of the eusocial species in Belize. Although based on only two collections, the pattern of declining colony size and increasing frequency of queenless colonies in Jamaica is strikingly similar to that observed in Belize (Fig. 4). *S. regalis* colony size declined from a median of 30 individuals in 2008 to five individuals in 2012, with about half the colonies queenless during both time periods. For *S. duffyi*, the pattern was similar: median colony size declined from 52 to four, and the percentage of colonies with a queen declined from 83 to 30 between 2008 and 2012.

## Discussion

Our results show that eusocial shrimp have declined greatly, and that three of the four eusocial species have apparently disappeared, from shallow reefs in the central Belize Barrier Reef after dominating these habitats for at least two decades. Prior to 2012 eusocial species were found on each of the nine sampling trips that spanned 14 years, and comprised 53–99% (median 87%) of the total numbers of shrimp collected in each year. Yet in 2012, only seven of 60 samples contained eusocial shrimp, all specimens of the host generalist *Synalpheus chacei*, which accounted for only 8% of shrimp collected in that year. Similar declines of eusocial species appear to be occurring in Jamaica.

A principal proximate reason for the decline of eusocial shrimp in Belize appears to be loss of the sponge species that host them. For example, *S. filidigitus* has been reliably recorded from three sponges in the Sand Bores: *Neopetrosia* (formerly *Xestospongia*, [20]) cf. *proxima*, *N*. cf. *subtriangularis*, and *Oceanapia* sp. [5]. *N*. cf. *proxima* in particular was moderately common prior to 2012, occurring in exactly half of the 20 samples from that period, whereas none of the 60 samples from 2012 contained this species. Sponges are a major group of space occupiers and play several key roles in coral reef ecosystems, including nutrient cycling [21]–[23] and, notably, provision of habitat for a diverse range of small animals [24]–[26]. Although less well-studied than corals, sponges have similarly declined steeply in many marine ecosystems, including Caribbean reefs, in recent decades [27]–[29]. These declines probably result from several causes, including worsening water quality and possibly increasing frequency of diseases [30]. A conspicuous case is the large Caribbean barrel sponge *Xestospongia muta*, which has shown increasing incidence of ‘sponge orange band disease’ in recent years [31]. The principle hosts of eusocial shrimp in Belize, namely *N.* cf. *proxima* and *N*. cf. *subtriangularis*, are in the same family as this species. If these cryptic sponges are similarly vulnerable to disease as their relative *X. muta*, this may help explain their loss from Belizean reefs in recent years.

Whether the disappearance of eusocial shrimp in Belize represents a long-term trend or a natural fluctuation in the reef community is unclear since quantitative time-series data are scarce for this and most other cryptofaunal reef taxa. But the decline occurs against a backdrop of disturbances that have fundamentally changed the world’s coral reefs in recent decades. These include top-down impacts of overfishing, spread of coral diseases, eutrophication, ocean acidification, and global climate warming [32]–[35]. In Belize specifically, corals have experienced rapid and tumultuous change in the last three decades [36]. After at least 3,000 years of stable dominance on shallow reefs, the staghorn coral *Acropora cervicornis* died out suddenly throughout the central Belize reef tract in the early 1980’s as a result of white band disease, and is now listed as critically endangered by the IUCN [37]. The lettuce coral (*Agaricia tenuifolia*) rapidly filled the void and came to dominance until it in turn died out catastrophically during a warm-water bleaching event in 1998 [38]. On deeper reefs in Belize, we have similarly observed decline in abundance of the coral *Madracis aurotenra* (formerly identified as *M. mirabilis*, [39]), dead branches of which are the major habitat of cryptic shrimp-bearing sponges in Belize. Such changes are not restricted to Belize: the long-dominant staghorn coral died out suddenly in Jamaica during the early 1980’s around the same time as it did throughout the Caribbean region [40].

The impacts of human-induced stressors on charismatic reef fauna such as corals and fishes are well recognized, but these organisms are only a small fraction of the biodiversity that reefs support [1], [41], [42]. The vulnerability of the large reservoir of cryptic biodiversity on tropical reefs has been little studied but is likely high, as our results hint. Specifically, the sponges inhabited by *Synalpheus* shrimp live primarily in rubble formed by dead branching corals, and the abundance and diversity of both sponges and associated shrimps varies greatly among rubble originating from different coral species. Thus, it’s likely that some of the changes in shrimp assemblages we observed arose from the documented succession of coral species in recent decades. By analogy, probabilistic modeling of several terrestrial systems of intimate associations predict that past extinctions of host organisms have resulted in numerous “coextinctions” of affiliated parasites, herbivores, and mutualists, and that host species currently listed as endangered also co-endanger >6000 species of their affiliates [43]. Given the rich biodiversity of animals that associate with sponges [25], [26], a similar situation is likely in the sea.

Finally, the decline and apparent disappearance of the most highly eusocial shrimp species in Belize and Jamaica –with colonies of hundreds of individuals and consistently only a single reproducing female–raise several questions about the link between eusocial organization and ecology. Attempts to explain the unique existence of eusociality in these marine animals have emphasized the proposed competitive advantage of cooperative social colonies [44], [45], and this hypothesis is consistent with comparative data from Belize showing greater abundance and host range in eusocial *Synalpheus* species than in sister taxa [6]. Although the preferential extinction of eusocial shrimp species in Belize appears to contradict the idea that eusocial species are competitively dominant, it may instead reflect vulnerability of the particular sponges favored by these host-specific animals. Regardless of the cause, the parallel declines of eusocial shrimp in Belize and Jamaica are cause for concern and suggest that change in Caribbean reef communities is deeper than what immediately meets the eye.

## Supporting Information

Figure S1 **Changes in shrimp community composition through time.** NMDS was performed on presence/absence data. Symbols as in Fig. 1
(TIF)

Table S1 **Composition of** ***Synalpheus*** **shrimp assemblages from the Sand Bores reefs, Belize between 1996 and 2012.**
(XLSX)

Table S2 **Composition of sponge assemblages from the Sand Bores reefs, Belize between 1996 and 2012.**
(XLSX)
