# Supplementary figures and images for: Decline and Local Extinction of Caribbean Eusocial Shrimp
Source: PLoS One. 2013 Feb 13;8(2):e54637. doi: 10.1371/journal.pone.0054637 (PMC3572134; doi:10.1371/journal.pone.0054637)

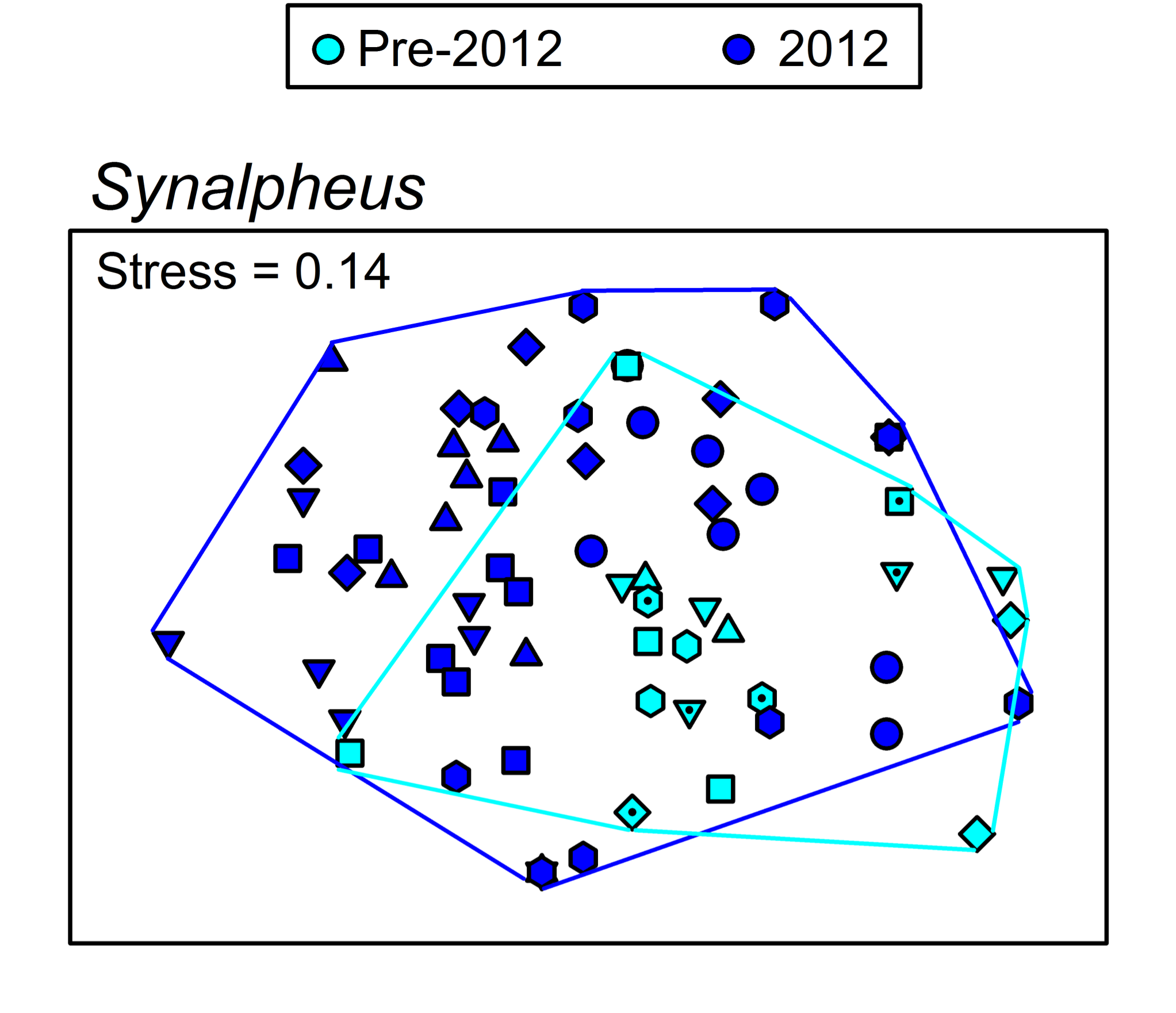

Supplement: Figure S1 — Changes in shrimp community composition through time. NMDS was performed on presence/absence data. Symbols as in Fig. 1 (TIF) [file pone.0054637.s001.tif]
